# Special Issue “Insect Viruses and Pest Management”

**DOI:** 10.3390/v12040431

**Published:** 2020-04-10

**Authors:** Miguel López-Ferber

**Affiliations:** LGEI, IMT Mines Ales, 30100 Ales, Occitanie, France; miguel.lopez-ferber@mines-ales.fr

Most revues consider the work on *Lymantria monarcha* in central Europe [1], at the end of the XIX century, as the starting point of virus control of insect pests. At that time, the viral nature of the infectious agent was unknown. During the early 1900s, many viruses were tested for the control of insect pests around the world, but it was only in 1970 when the first virus-based insecticide was registered in the USA to control the cotton bollworm [2]. Since then, the use of many viruses to control insect pests has been authorized, and increasing research efforts are devoted to the characterization of new viruses and the evaluation of their potential.

Two previous Special Issues addressing insect viruses have been published in *Viruses*: the first on 2011, edited by Dawn Gundersen-Rindal and Robert L. Harrison; the second in 2015, edited by John Burand and Madoka Nakai.

In 2011, the Issue covered all aspects of insect viruses. Among the contributions, a review paper discussed the future importance of massive sequencing for the discovery of new insect viruses [3].

In 2015, the Special Issue was entitled “Insect viruses and their use for microbial pest control”. It presented 10 contributions, including two reviews on the use of viruses for the control of insect pests in Latin America [4] and China [5], as well as three research articles on the use of two viruses in the field [6,7,8], confirming the expanding use of this approach.

The increasing questioning of the negative environmental impacts of agriculture promoted the promulgation of objectives of reducing chemical insecticide use. One of the suitable alternatives is biological control, and viruses have proven their efficacy. This is the second Special Issue concerning the use of insect viruses in pest control.

In this Issue, 20 contributions are published. The majority of these contributions address the potential of the virus to fight insect pests, but some consider the importance of the viruses of beneficial insects (honey bees). The generalization of massive sequencing confirmed that multiple infections are more common than previously expected. Viruses remain in host populations for a long time without apparent effect on the hosts (covert infections).

The field resistance of codling moth to specific CpGV genotypes highlighted the importance of genotypic diversity in the virus populations and the role of multiple infections, which are addressed in various contributions. Taking advantage of this diversity might be one of the keys to ensuring the long-term efficiency of virus control.

I hope the reviews and research articles of this Special Issue will fruitfully contribute to developing the knowledge and use of insect viruses in pest control.
